# α-Mangostin Is a Xanthone Derivative from Mangosteen with Potent Immunomodulatory and Anti-Inflammatory Properties

**DOI:** 10.3390/biom15050681

**Published:** 2025-05-07

**Authors:** Amin F. Majdalawieh, Bayan K. Khatib, Tala M. Terro

**Affiliations:** 1 Department of Biology, Chemistry and Environmental Sciences, College of Arts and Sciences, American University of Sharjah, Sharjah P.O. Box 26666, United Arab Emirates; g00095426@aus.edu (B.K.K.); g00086819@alumni.aus.edu (T.M.T.); 2 Advanced Biosciences and Bioengineering Research Center, American University of Sharjah, Sharjah P.O. Box 26666, United Arab Emirates

**Keywords:** α-mangostin, inflammation, xanthone derivative, oxidative stress, NF-κB signaling pathway

## Abstract

α-Mangostin, a bioactive xanthone derived from the *Garcinia mangostana* L. Clusiaceae (*G. mangostana*) fruit, has demonstrated significant anti-inflammatory and immunomodulatory properties. Chronic inflammation plays a critical role in the pathogenesis of various diseases, including metabolic disorders, autoimmune conditions, and cancer. Conventional anti-inflammatory therapies, such as non-steroidal anti-inflammatory drugs (NSAIDs), often carry undesirable side effects, prompting the need for safer, natural alternatives. This review consolidates the existing literature on the mechanisms by which α-mangostin exerts its anti-inflammatory effects, including the suppression of pro-inflammatory cytokines, modulation of immune cell activity, and inhibition of key signaling pathways such as nuclear factor-kappa B (NF-κB) and mitogen-activated protein kinase (MAPK). Additionally, α-mangostin exhibits immunomodulatory properties by influencing both innate and adaptive immune responses, affecting macrophage polarization, T cell differentiation, and cytokine production. Its efficacy has been observed in numerous disease models, including joint disorders, digestive and metabolic conditions, hepatic diseases, neurological disorders, and respiratory ailments. The potential therapeutic applications of α-mangostin as an anti-inflammatory agent warrant further investigation through preclinical and clinical studies to validate its efficacy and safety.

## 1. Introduction

Inflammation is an important part of the tissue response to harmful inflammatory agents [1]. Inflammation can be caused by pathogenic factors, which can be infectious or non-infectious (physical, chemical, biological, or psychological) factors [2]. This response involves numerous inflammatory cells and cytokines. In the case of microbial infections, components of the innate immune system, the toll-like receptors (TLRs) and NOD (nucleotide-binding oligomerization domain protein)-like receptors (NLRs), are activated and recognize an infection, leading macrophages and mast cells to produce chemokines, cytokines, vasoactive amines, eicosanoids, and products of proteolytic cascades to allow leukocytes like neutrophils to eliminate the invading agent by releasing toxic substances like reactive oxygen species (ROS) and reactive nitrogen species (RNS) at the site of infection [3]. Chronic inflammation results when these mechanisms responsible for mediating inflammation are not regulated, and it is linked with metabolic disorders, allergies, autoimmune disorders, and even the development of cancer because of overactive neutrophil infiltration and an abundance of ROS, RNS, and tissue-damaging enzymes [4]. Tissue damage can also result from free radicals, namely nitric oxide (NO), that can cause toxic oxidative reactions [5]. As a result, anti-inflammatory drugs, like non-steroidal anti-inflammatory drugs (NSAIDs), have been developed and are often used for the treatment of these inflammatory disorders [6]. Most NSAIDs work by inhibiting the cyclooxygenase (COX) enzyme that synthesizes eicosanoids like prostaglandins [7]. However, NSAIDs also have the potential risk of producing many adverse side effects, including gastric, renal, cardiovascular, hepatic, and hematologic effects [8]. As a result, many researchers have been investigating natural alternatives to manage chronic diseases to counter the side effects and prices of existing treatments [1]. For instance, previous reviews have investigated the immunomodulatory and anti-inflammatory effects of natural lignans like Nigella sativa and thymoquinone [5], berberine [9], sesamin [6], and sesamol [10]. Natural products such as cyanidins [11] and sesamol [12] have also been investigated for their anti-cancer properties.

**Figure 1:**
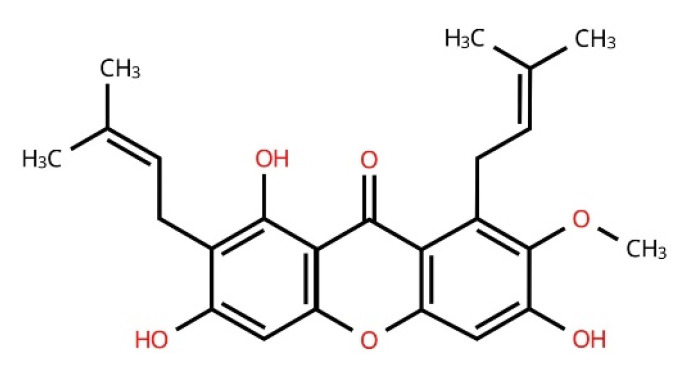
Chemical structure of α-mangostin.

The xanthone α-mangostin is a secondary metabolite from the mangosteen fruit, native to Southeast Asia [13]. Valued for its flavor and traditional medicinal uses, mangosteen is a rich source of carbohydrates, fiber, vitamin C, folate, and potassium. Its pericarp contains a high concentration of bioactive compounds, particularly xanthones such as α-mangostin, γ-mangostin, garcinone E, flavonoids, tannins, saponins, and polysaccharides. The efficient extraction of α-mangostin has been achieved through several methods. Conventional solvent extraction using ethanol or ethyl acetate is common, but often requires additional purification. Ultrasound-assisted extraction (UAE) improves yield and efficiency under optimized conditions (70–80% ethanol, 40–50 °C, 30 min), while microwave-assisted extraction (MAE) offers rapid recovery within minutes using similar solvent concentrations [13]. Supercritical fluid extraction (SFE) with CO_2_ and ethanol provides high purity and environmental safety, though it demands more sophisticated equipment. Post-extraction purification typically involves liquid–liquid extraction, silica gel column chromatography, and preparative high-performance liquid chromatography (Prep-HPLC), ensuring pharmaceutical-grade α-mangostin suitable for therapeutic development [13]. α-Mangostin has a molecular formula of C_24_H_26_O_6_ and a molecular weight of 410.45 g/mol. The compound is known for its poor solubility in water but is soluble in organic solvents such as ethanol, methanol, and dimethyl sulfoxide (DMSO). With a melting point of around 180–181 °C, the chemical properties of α-mangostin play a crucial role in its bioactivity, especially in the context of therapeutic applications. The xanthone structure of α-mangostin significantly contributes to its bioactivity through its unique chemical configuration, which comprises a tricyclic aromatic system with various functional groups attached to its rings (Figure 1). This structural arrangement facilitates interactions with biological targets, influencing its pharmacological activities. The specific functional groups at positions C-1, C-3, C-6, and C-8 of the xanthone molecule are crucial in defining the biological activity of α-mangostin. These positions are implicated in modulating various pathways, such as anti-inflammatory and anti-cancer mechanisms. The presence of hydroxyl groups, in particular, enhances antioxidant capacity, allowing α-mangostin to scavenge free radicals effectively, thereby exerting cytoprotective effects. While α-mangostin demonstrates a favorable safety profile with no apparent hepatotoxic effects at common dosages, interactions with certain medications may pose risks. Thus, individuals on specific drug therapies should approach its use with caution and under medical supervision to mitigate any potential adverse effects. Mangosteens are known as a “super fruit” and have been traditionally used to treat diarrhea, skin infections, inflammation, fever, and cholera [14]. α-Mangostin has been shown in previous studies to have anti-cancer [15], antioxidant [16], and analgesic [17] effects. Recently, we extensively reviewed the anti-cancer effects of α-mangostin in various types of cancer [18]. Numerous in vitro and in vivo experimental studies have been conducted on this xanthone’s anti-inflammatory and immunomodulatory effects. However, to date, no comprehensive review has compiled these anti-inflammatory and immunomodulatory effects and their underlying mechanisms. Therefore, this review aims to compile its anti-inflammatory effects in general and in various organ-related diseases, its cellular and humoral immunomodulatory effects, and the signaling pathways involved in modulating these effects in order to better understand α-mangostin as a potential treatment for inflammatory diseases.

## 2. Search Methodology

The literature search was conducted using a variety of online databases, such as PubMed and Google Scholar. To ensure the identification of relevant studies, the word “α-mangostin” was used along with keywords including “inflammation”, “cytokines”, and “immunomodulation”. Articles were screened and selected, with no restriction on publication date, the experimental subjects/models used, the mode and duration of α-mangostin administration, or other experimental details.

## 3. Anti-Inflammatory Effects of α-Mangostin

Inflammation is a spontaneous natural response of tissues and organs as a response to acute injuries, chronic injuries, or pathogenic infections [19]. Acute inflammation is a transient response that efficiently eliminates the damaging agent to allow tissue repair through the release of various molecular processes [4]. Major inflammatory markers, or cytokines, that can be found in serum include interleukin (IL)-8, IL-1β, IL-6, and tumor necrosis factor (TNF) [19]. However, when inflammation is maintained, chronic inflammation can result, leading to healthy tissue damage via DNA lesions [4]. During chronic inflammation, tissues can produce oxygen and nitrogen radicals, activate the transcription factor nuclear factor-kappa B (NF-κB), and accumulate excess cytokines like TNFα and IL-6 that are associated with and can lead to cancer [19]. The cancer-inducing potential of inflammation and existing inflammatory diseases facilitate the need for the discovery of more anti-inflammatory drugs and molecules, potentially in the form of natural products like α-mangostin.

In an in vitro study by Gutierrez-Orozco et al. (2013), the dose-dependent anti-inflammatory effects of α-mangostin were investigated in the following cell lines: RAW 264.7 (macrophage-like cells), MDM cells (monocyte-derived macrophages), THP-1 (monocyte-like leukemia cells), HepG2 (hepatocellular carcinoma cells), Caco-2 HTB-37 (enterocyte-like colorectal adenocarcinoma cells), and HT-29 (colorectal adenocarcinoma cells) [20]. α-Mangostin (1, 3, and 10 μM) was found to attenuate the lipopolysaccharide (LPS)-induced production of NO by 50%, 61%, and 78%, respectively, in RAW 264.7 macrophages. It was found that the inhibitory effects of α-mangostin for the secretion of pro-inflammatory mediators depend on the cell line type and cellular activation [20]. Pretreatment with α-mangostin (7 μM) significantly reduced the release of TNFα by phorbol myristate acetate (PMA)-stimulated HepG2 cells. Additionally, α-mangostin (10 μM) reduced IL-8 secretions by LPS-stimulated THP-1 and colonic HT-29 cells, and α-mangostin (15 μM) reduced IL-8 secretions by enterocyte-like Caco-2 cells. Alternatively, the α-mangostin (4.5 μM) pretreatment of MDM cells raised TNFα secretion more than 50% in response to LPS stimulation, and in un-stimulated cultures, α-mangostin (4.5 and 10 μM) also more than doubled the basal secretion of TNFα by MDM cells and IL-8 by HT-29 cells, indicating pro-inflammatory activity during cellular inactivation [20]. Researchers speculated that these immunomodulatory observations were due to alterations in cellular metabolism and the increased bioavailability of α-mangostin under inflammatory conditions [20]. In another in vitro study by Tewtrakul et al. (2009), the anti-inflammatory effects of mangosteen fruit hull, α-mangostin, and γ-mangostin from the extract of *G. mangostana* were tested using the RAW 264.7 macrophage cell line [21]. *G. mangostana* extract and the isolated α-mangostin and γ-mangostin compounds at concentrations ranging from 3 to 100 μg/mL were investigated against LPS-induced NO, prostaglandin E2 (PGE2), TNFα, and IL-4. There was an inhibitory effect on NO release with a half-maximal inhibitory concentration (IC_50_) value of 1.0 μg/mL for *G. mangostana* extract, 3.1 μM for α-mangostin, and 6.0 μM for γ-mangostin. There was also an inhibitory effect on PGE2 release with an IC_50_ value of 6.0 μg/mL for *G. mangostana* extract, 13.9 μM for α-mangostin, and 13.5 μM for γ-mangostin. The extract had more potent effects in inhibiting NO and PGE2 than the isolated compounds. There were only moderate effects on TNFα and IL-4 release, with IC_50_ values between 31.8 and 64.8 μM for α-mangostin and γ-mangostin. Additionally, using mRNA expression assays, treatment with the extract, α-mangostin (3, 10, and 30 μM), and γ-mangostin (3, 10, and 30 μM) for 20 h all suppressed gene transcription for inducible nitric oxide synthase (iNOS), while only the extract and α-mangostin inhibited cyclooxygenase-2 (COX-2) transcription. In another in vitro study, the role of α-mangostin against LPS (10 μg/mL)-induced inflammation was studied in the intestinal epithelial cell (IEC-6) line [22]. Pretreating the cells with α-mangostin (2.5, 5, and 10 μM) caused a dose-dependent decrease in the production of an array of LPS-induced cytokines, namely NO, PGE2, IL-6, TNFα, and IL-1β [22]. Similarly, α-mangostin (2.5, 5, and 10 μM) also dose-dependently inhibited the LPS-induced mRNA expressions of the enzymes iNOS and COX-2; the cytokines IL-6, TNFα, and IL-1β; and the innate immunity-related receptor and adaptor proteins TLR4 and myeloid differentiation primary response 88 (MYD88), involved in the TLR signaling pathways, in IEC-6 cells. These results suggest that α-mangostin suppresses pro-inflammatory cytokine release in inflammatory intestinal diseases through inhibiting the activation of TLR4-mediated transforming growth factor beta-activated kinase 1 (TAK1)-NF-κB signaling pathways [22]. Similarly, one combined in vitro and in vivo study assessed the anti-inflammatory properties of α-mangostin in LPS-induced IEC-6 cells and in inflammatory bowel disease (IBS)-modeled male Sprague Dawley rats [23]. α-Mangostin (10 μM) administration significantly downregulated LPS-elevated mRNA and the protein expression of NOD-like receptor protein 3 (NLRP3), caspase 1, IL-1β, and IL-18 in IEC-6 cells. In mice, chemokine CXC ligand (CXCL)17 and IL-23α, both of which were recently found to have anti-inflammatory effects, were also elevated in the α-mangostin (50 mg/kg) treatment group [23].

α-Mangostin has also been studied regarding more specific organ- or pathogen-related inflammation. One in vivo study examined the protective potential of α-mangostin against UVB damage in HR-1 hairless male mice and found that α-mangostin (100 mg/kg) inhibited UVB-induced wrinkle formation and increased epidermal thickness more than 2-fold when compared to nontreatment groups [24]. Additionally, α-mangostin also exhibited antioxidant effects, through enhancing superoxide dismutase (SOD) catalytic activity, and overall photoprotective effects, through the suppression of pro-inflammatory cytokine mRNA expression, including IL-1β, IL-6 and TNFα, and the protein expression of matrix metalloproteinase (MMP)-1 and -9. Numerous studies have linked the pathogenesis of photoaging to MMP induction and cytokine release [24]. Overall, Im and colleagues effectively demonstrated α-mangostin’s potential in alleviating UVB-induced skin damage. Other studies, like the following in vitro experiments, examined the inhibitory effect of α-mangostin on dengue virus (DENV) expression [25,26,27]. Treating HepG2 cells with α-mangostin (5, 10, and 20 μM) for 24 h [26] and human hepatocellular carcinoma (Huh)-7 cells with α-mangostin (10, 15, and 20 μM) for 24, 48, and 72 h [27] resulted in a dose-dependent inhibition of DENV infection. This was also shown in the study by Sugiyanto et al. (2019), where α-mangostin (10 and 20 μM) treatment for 24 and 48 h in human peripheral blood mononuclear cells (PBMCs) resulted in more than 50% viral reduction, with a greater reduction effect at 48 h [25]. Tarasuk et al. (2022) showed that the production of DENV-2-infected cells decreased to 35% with α-mangostin (20 μM) [26]. They also found that α-mangostin downregulated the expression of all DENV-2 proteins and that α-mangostin inhibited the DENV life cycle by reducing the viral replication step. Additionally, using an in vitro fluorescence-based transcription assay, a dose–response curve plotted for α-mangostin (0–40 μM) showed an IC_50_ value of 16.50 μM for inhibiting DENV-2 RNA-dependent RNA polymerase (RdRp) activity. Tarasuk and colleagues (2017) also found the transcription of these cytokines and chemokines to be reduced in DENV-1, DENV-3, and DENV-4-infected cells, with α-mangostin reducing them more in DENV-4 than ribavirin and dexamethasone. Additionally, the Sugiyanto et al. (2019) study showed that α-mangostin treatment (5, 10, and 20 μM) for 24 and 48 h reduced TNFα and IFNγ expression, with higher reduction rates at higher α-mangostin concentrations. α-Mangostin also has the potential to alleviate multi-organ-involved conditions. For example, in a combined in vitro and in vivo study, the ability of α-mangostin to suppress the macrophage-mediated host response to LPS-induced inflammation and cecal ligation and puncture (CLP) induction in BALB/c mice was explored. In mice murine peritoneal macrophages, α-mangostin (10, 20, 40, and 80 μM) treatment for 24 h reversed the LPS-induced increase in NLRP3 inflammasome activation, including the mRNA and protein levels of NLRP3, ASC, caspase1, and IL-1β [28]. Studies suggest that NLRP3 inflammasome activation is associated with immune dysfunction and sepsis-induced multiple organ injury [29,30,31]. In vivo, α-mangostin (3 mg/kg) pretreatment suppressed circulating levels of TNFα, IFNγ, and IL-1β, as well as the hepatocellular injury markers ALT and AST, whilst also upregulating levels of IL-10 in CLP-induced septic mice. Altogether, these results suggest α-mangostin as a protective agent against sepsis and as a negative regulator of CLP-induced inflammation and vital organ injuries [28].

### 3.1. Joint Disorders

There are numerous studies displaying the therapeutic benefits of α-mangostin for joint disorders like arthritis. For example, in one combined in vitro and in vivo study, the effects of α-mangostin in alleviating angiogenesis in adjuvant-induced arthritic (AIA) male Wistar rats and human umbilical vein endothelial cells (HUVECs) were examined [32]. α-Mangostin (30 mg/kg) treatment in AIA rats restored elevated blood levels of granulocytes, lymphocytes, and intermediate cells, shown by decreased paw swelling and joint deformation. AIA- and LPS-induced elevated serum levels of angiogenic and inflammatory mediators, such as IL-6 and transforming growth factor-β (TGF-β), were also restored by α-mangostin (30 mg/kg) treatment. AIA-induced hypoxia-inducible factor-1α (HIF-1α), vascular endothelial growth factor (VEGF), and LPS-induced COX-2 levels were also found to be effectively suppressed after α-mangostin administration. Specifically for VEGF, the effects were also observed in synovium tissues. Additionally, the α-mangostin (2, 4, and 6 μg/mL) treatment of LPS-primed HUVECs resulted in a dose-dependent reduction in VEGF production, as well as a reduction in VEGF-induced HUVEC migration into wounds [32]. Another combined in vitro and in vivo study explored the therapeutic effects of α-mangostin in rats with AIA and human fibroblast-like synoviocytes (HFLSs) rheumatoid arthritis (RA) cells [33]. α-Mangostin (40 mg/kg) given once per day for 35 days by intragastric administration protected the destruction of joints and showed anti-inflammatory activity in the initial stages of AIA as α-mangostin reduced inflammatory leucocytes, neutrophil, lymphocyte, and platelet cells. α-Mangostin treatment (40 mg/kg) also reduced the expression of the inflammatory cytokines TNFα and IL-β [33]. In another combined in vitro and in vivo study, the anti-rheumatic and fat metabolism effects of α-mangostin were investigated in AIA-induced male Sprague Dawley rats and pre-adipocytes [34]. Treatment with α-mangostin (50 mg/kg) for 30 days increased lymphocyte and hemoglobin counts and restored the expression of nicotinamide phosphoribosyltransferase (NAMPT), SIRT1, and peroxisome proliferator-activated receptor (PPAR)-γ, indicating its anti-inflammatory properties and promising profound metabolic changes. Glucose and total triglyceride levels in peripheral blood, which were reduced in AIA rats during the peak of secondary inflammation, were also reversed by α-mangostin (50 mg/kg) treatment. Elevated AIA-induced serum levels of IL-1β and IL-6, involved in white adipose tissue (WAT)-related inflammation, were also suppressed by α-mangostin (50 mg/kg) treatment. Moreover, the indicators of oxidative stress in AIA rats also saw considerable alterations via decreased SOD activity, while treatment with α-mangostin (50 mg/kg) reversed this modification. α-Mangostin (4 μg/mL) treatment for 24 h also upregulated the expression of the anti-inflammatory mediators SIRT1 and PPARγ in pre-adipocytes. Moreover, pre-adipocyte co-culture dramatically exacerbated LPS-induced inflammation, shown by elevated levels of IL-1β, IL-6, and monocyte chemoattractant protein (MCP)-1. α-Mangostin (4 μg/mL) therapy, however, suppressed this rise in pro-inflammatory cytokines. Pre-adipocytes supported the upregulation of IL-1β and iNOS and its upstream TLR4, COX-2, p-JNK, and p-p65 in LPS-treated macrophages, which were all also downregulated by α-mangostin. [34]. Additionally, a combined in vitro and in vivo study also proved α-mangostin to be successful in attenuating collagen-induced RA in male Sprague Dawley rats and LPS-induced HFLSs through the suppression of NAMPT [35]. Orally treating rats with α-mangostin (40 mg/kg) suppressed the synovium expression of NAMPT and serum levels of NAD^+^ compromising the salvage biosynthesis of NAD. Similarly, treatment with α-mangostin (8, 10, and 12 μg/mL) for 6 h, but not 12 h, suppressed NAMPT and NAD levels in HFLSs. Histological examinations proved α-mangostin (40 mg/kg) to alleviate the collagen-induced matrix degradation of the joints, inflammatory cell infiltration, and synovial hyperplasia [35].

Furthermore, in another combined in vitro and in vivo study, the potential for the inhibition of osteoarthritis (OA) progression was examined in male Sprague Dawley rats and their chondrocytes [36]. Western blots proved α-mangostin (3, 6, and 12 μM) treatment for 2 h to dose-dependently alleviate the IL-1β-induced downregulation in collagen II, Aggrecan, and their regulatory factor, SOX-9, and the upregulation of MMP-13 and ADAMTS-5 in chondrocytes. MMP-13 and ADAMTS-5 are also enzymes known for the catalysis of components of the cartilage matrix [37]. Additionally, in a surgically induced OA rat model, α-mangostin (10 mg/kg) treatment for 8 weeks morphologically reduced matrix degradation, articular cartilage thickness, and cartilage damage. Overall, researchers suggested that the anti-OA potential of α-mangostin may lie in its ability to regulate catabolic factors [36]. Similarly, in another combined in vitro and in vivo study, α-mangostin was investigated for its protective effects against IL-β-induced inflammation in male Sprague Dawley OA rat models and their chondrocytes [38]. In addition to the cytotoxic effects of α-mangostin found via CCK-8 assay, α-mangostin (1.25, 2.5, 5.0, and 10 μg/mL) treatment for 24 h decreased IL-1β-induced PGE2, NO, p-p65, and p-IκB expressions in a dose-dependent manner in rat chondrocytes. Moreover, pretreating IL-1β-stimulated chondrocytes with α-mangostin dose-dependently inhibited the release of iNOS, COX-2, MMP-3, MMP-9, and MMP-13. Treating IL-1β-stimulated chondrocytes with α-mangostin (5.0 μg/mL) also reduced the activation of NF-κB p65 nuclear translocation, alleviating joint inflammation and OA progression in rats. α-Mangostin (1.25, 2.5, and 5.0 μg/mL) was also shown to increase the expression of collagen II and Aggrecan in a dose-dependent manner. Furthermore, chondroprotective effects, which increase articular cartilage thickness and ameliorate cartilage damage, were observed in rat OA models with α-mangostin (10 mg/kg) administration [38]. In another combined in vitro and in vivo study, to study therapeutic effects against RA, the anti-inflammatory effects of α-mangostin were investigated in collagen-induced arthritic (CIA) male DBA/1J mice and mouse-derived bone marrow dendritic cells (mBMDCs) [39]. Initially, arthritic scores were used to gauge the therapeutic potential of α-mangostin. α-Mangostin (10 and 40 mg/kg) showed a significant decrease in arthritic score from day 7 to 18 of treatment compared to the vehicle- and methotrexate-treated groups [39]. Histological scores showed the effectiveness of α-mangostin treatment for 33 days in managing joint cartilage inflammation in mice knees to be significant only at 40 mg/kg. The CIA-induced mice showed higher inflammatory cell infiltration, like mononuclear cells and fibroblasts, whereas α-mangostin treatment reduced their scores [39]. Moreover, vehicle-elevated PGE2 levels were suppressed in a dose-dependent manner with α-mangostin and methotrexate treatment in serum and in the joints, with the latter showing superior effects. None were significantly effective, however [39]. Regarding CIA-induced elevated joint inflammatory mediators, α-mangostin treatment dose-dependently suppressed the production of IL-6, CXCL5, CXCL10, and CCL5, while α-mangostin (40 mg/kg) also suppressed CXCL9 levels [39]. Additionally, both doses of α-mangostin and methotrexate treatments markedly downregulated IL-33 expression in joint tissues, compared to that of the vehicle-induced mice [39]. Moreover, α-mangostin (3 and 5 μg/mL) treatment of LPS-stimulated mBMDCs was found to notably reduce IL-12 and increase IL-10 production, respectively, suggesting the anti-inflammatory effects of α-mangostin, while the levels of IL-6, TNFα, and TGF-β remained unchanged [39]. Two more combined in vitro and in vivo studies articulated the therapeutic effects of α-mangostin through the activation of the choline anti-inflammatory pathway (CAP) in collagen-induced [40] and adjuvant-induced [41] RA in male Sprague Dawley rats, PBMCs, and RAW 264.7 cells. Compared to controls, α-mangostin (40 mg/kg) treatment ameliorated the inflammatory severity in arthritic rats, including inflammatory cell infiltration, articular cartilage erosion, and splenic lymphocyte density around arteries [40,41]. Moreover, α-mangostin (40 mg/kg) simulated the CAP by restoring the collagen- and adjuvant-induced decline in α7nAChR expression, while simultaneously markedly downregulating the circulating levels of IL-β and TNFα. Additionally, in lymphocyte-dominated PBMCs, α-mangostin (5 μg/mL) attenuated the catalytic activity of AChE, leading to the accumulation of ACh, allowing solid conclusions to be drawn towards α-mangostin-led CAP activation [40]. Finally, Chen et al. (2022) also found that α-mangostin (5 μg/mL) reversed adjuvant-suppressed SIRT1 expression in RAW 264.7 macrophages, drawing a link between energy metabolism and immune inflammation [41]. Overall, these studies demonstrated the ability of α-mangostin to ameliorate the peripheral immune environment through CAP activation [40]. Overall, this extensive number of studies provides support for the anti-inflammatory benefits of α-mangostin in arthritis.

### 3.2. Digestive and Metabolic Disorders

α-Mangostin has been studied for its anti-inflammatory benefits in digestive and metabolic disorders. In one in vivo study, researchers investigated the effects of α-mangostin in ameliorating dextran sulfate sodium (DSS)-induced ulcerative colitis (UC) in male ICR mice [42]. Oral pretreatment with α-mangostin (30 mg/kg) for 7 days suppressed DSS-stimulated rises in serum levels of ROS, NO, and myeloperoxidase (MPO), exhibiting significant antioxidative and anti-inflammatory effects. The enzyme MPO is widely generated by macrophages, monocytes, and neutrophils. MPO activity is thought to be a particular marker of neutrophil infiltration in colitis, which causes inflammatory damage to the mucosa [43]. Compared to the control group, α-mangostin (30 mg/kg) pretreatment also completely restored MCP-1 colonic mRNA expression, while also succeeding at downregulating TLR-2 mRNA expression [42]. Additionally, α-mangostin (30 mg/kg) also significantly suppressed the DSS-stimulated colonic levels of TNFα mRNA expression, but did not, however, restore these levels, as seen with sulfasalazine (100 mg/kg) and *G. mangostana* pericarp extract (40, 200, and 1000 mg/kg) treatments. Moreover, it is suggested that pro-inflammatory cytokines, including TNFα, increase the expression of adhesion molecules. In this regard, qPCR analysis showed α-mangostin (30 mg/kg) pretreatment to completely restore the DSS-upregulated colonic levels of the intracellular adhesion molecule ICAM-1, while significantly suppressing those of vascular cell adhesion molecule (VCAM)-1 [42]. Similarly, another in vivo study by You et al. (2017) demonstrated the effects of α-mangostin in alleviating DSS-induced colitis in 6-week-old mice by inhibiting the inflammatory NF-κB and MAPK pathways [44]. The disease activity index (DAI) scores were lower in both the DαM30 (30 mg/kg of α-mangostin) and DαM100 (100 mg/kg of α-mangostin) group than in the DSS group. After dissection, the colon lengths of both treatment groups, DαM30 (9.57% increase) and DαM100 (21.5% increase), were significantly longer than the DSS group, which was shorter than the control by 37.5%. MPO levels in the esophagi and colons increased in the DSS group, but this was reversed in the 30 and 100 mg/kg treatment groups, indicating the inhibition of leukocytes. Surface epithelial cell damage, inflammatory cell infiltration, and submucosal edema induced by DSS was attenuated by the 100 mg/kg treatment, but only partly by the 30 mg/kg treatment [44]. Collectively, these results validate the anti-inflammatory potential of α-mangostin in the development of novel UC therapies. The following in vivo study investigated the impact of a diet containing α-mangostin on colitis induced by DSS in mice [45]. In contrast to the previous studies, the DAI was higher in the group that received both DSS and α-mangostin compared to the DSS-only group. The DSS + α-mangostin group showed more severe symptoms, including greater weight loss, diarrhea, and rectal bleeding. Inflammation and crypt injury were also more severe in the mid and distal colon of the DSS + α-mangostin group after treatment cessation. While food intake decreased temporarily in the DSS + α-mangostin group, it normalized after five days, with no liver toxicity observed. Microscopic analysis confirmed higher inflammation, crypt injury, and ulceration in the DSS + α-mangostin group. Colonic epithelial cell proliferation, as measured by Ki67 expression, and the infiltration of T cells and macrophages were greater in the DSS + α-mangostin group compared to DSS-only and control groups. Hyperplasia was also more pronounced in the DSS + α-mangostin group, persisting even after two weeks of recovery. The study by Gutierrez-Orozco and colleagues also analyzed the effects of α-mangostin on neutrophil infiltration, systemic inflammation, and gut microbiota in DSS-induced colitis in mice. MPO levels, an indicator of neutrophil activity, were significantly higher in the DSS + α-mangostin group compared to DSS-only and control groups. Splenomegaly and increased liver weight were observed in the DSS + α-mangostin group, along with elevated levels of systemic inflammatory markers, including SAA, IL-6, and granulocyte colony-stimulating factor [45]. This study, unlike the first two mentioned, provides support against α-mangostin as a potential treatment for colitis, suggesting that more in vivo studies should be performed to clarify the difference in results.

Another extensive combined in vitro and in vivo study investigated α-mangostin for its ameliorating effects against LPS-induced and age-related adipose tissue inflammation in young and old male C57BL/6J mice and their RAW 264.7 macrophages [46]. α-Mangostin (10 mg/kg) administration for 5 days reduced LPS-stimulated inflammatory cytokine levels in serum and epididymal white adipose tissue (eWAT). α-Mangostin (5 μM) pretreatment also decreased inflammatory cytokines in LPS-stimulated RAW 264.7 cells. These reductions included IL-6, TNFα and MCP-1 [46]. Similar results were found in old mice, with the addition of decreased IL-1β and COX-2 levels after α-mangostin (25 and 50 mg/kg) treatment. Reduced overall eWAT inflammation is thought to decrease the incidence of insulin resistance and metabolic disorders [47]. Additionally, LPS-elevated levels of iNOS, the key enzymatic mediator of inflammatory NO production [48], were reversed by α-mangostin (10 mg/kg) in vivo and α-mangostin (5 μM) in vitro [46]. Compared to untreated old mice, α-mangostin (25 and 50 mg/kg)-treated old mice showed significant decreases in triglycerides, LDL-C, total cholesterol, AST, and ALT hepatic levels [46]. Interestingly, a type of macrophage-specific inflammatory RNA, microRNA-155-5p (miR-155), was also suppressed after α-mangostin (25 and 50 mg/kg) treatment in the serum and eWAT of old mice, as well as after α-mangostin (5 μM) pretreatment in LPS-stimulated RAW 264.7 macrophages [46]. Through this study, α-mangostin proved to ameliorate adipose tissue inflammation, which could be used therapeutically in metabolic disorders. Additionally, an in vitro study evaluated α-mangostin’s ability to prevent LPS-mediated metabolic inflammation and insulin resistance in human primary adipocytes compared to its isomer, γ-mangostin [49]. α-Mangostin (3 μM) successfully attenuated the LPS-induced mRNA expression of several pro-inflammatory mediators, including TNFα, IL-1β, IL-6, IL-8, MCP-1, and TLR-2, while γ-mangostin did so more effectively equimolarly. Another in vitro study by Bumrungpert et al. (2010) found that the α-mangostin (3, 10, and 30 μM) treatment of LPS-induced Human U937 monocytes also dose-dependently attenuated interferon (IFN) y-inducible protein (IP)-10 [50]. Moreover, researchers reported only γ-mangostin inhibiting the LPS suppression of PPARγ and adiponectin mRNA levels in adipocytes, while both isomers successfully did so in human macrophages [50], establishing a link between WAT inflammation and insulin resistance, calling for further research on the effects of α-mangostin in this regard [49]. Although limited in number, data from these studies show α-mangostin’s potential in alleviating metabolic impairments, and further experimental studies should be performed to more comprehensively see its effects.

### 3.3. Hepatic Disorders

Previous studies have also examined α-mangostin for its preventative and therapeutic anti-inflammatory properties in liver diseases. For instance, in a combined in vitro and in vivo study, the anti-inflammatory effects of α-mangostin were investigated in high-fat-diet-induced C57BL/6 mice for 12 weeks [51]. α-Mangostin (50 mg/kg/day) showed reduced macrophage infiltration and inflammatory cytokines in the liver and white adipose tissue (WAT) of α-mangostin-treated mice compared to high-fat diet (HFD) control mice. This was shown using immunohistochemistry with the macrophage marker F4/80. Additionally, the study examined the expression of pro-inflammatory cytokines (TNFα, MCP-1, CCR2, and IL-6) and the anti-inflammatory cytokine IL-10 in the liver, WAT, and LPS-treated RAW 264.7 cells. The expression of pro-inflammatory mediators (e.g., MCP-1 and IL-6) were notably reduced in the HFD/α-mangostin mice compared to the HFD control mice, while IL-10 expression remained unchanged in WAT and increased in the liver and LPS-treated RAW 264.7 cells [51]. These findings suggest that α-mangostin (50 mg/kg/day) may mitigate HFD-induced inflammation in WAT by modulating macrophage infiltration and altering the expression of pro-inflammatory cytokines. In another in vivo study, researchers studied the hepatoprotective effects of α-mangostin against liver-fibrosis-inducing thioacetamide (TAA) in male Wistar rats [52]. In the α-mangostin (50 mg/kg) and α-mangostin + TAA treatment groups, the hepatic inflammation markers AST and ALT were markedly lower than in the TAA-only group. Additionally, immunohistograms showed that the co-administration of α-mangostin (50 mg/kg) and TAA significantly downregulated hepatic tissue levels of TGF-β1, α-SMA, and TIMP-1; however, the α-mangostin (50 mg/kg) group alone only did so for TIMP-1, hinting at its protective effects against hepatic inflammation [52]. Likewise, one in vivo study by Fu et al. (2018) showed that α-mangostin (12.5 and 25 mg/kg) has hepatoprotective effects on lipopolysaccharide/d-galactosamine (LPS/D-GalN)-induced acute liver failure in mice [53]. Mice pretreated with α-mangostin (12.5 and 25 mg/kg) for 7 days showed significantly decreased AST and ALT levels from the LPS/D-GalN treatment. α-Mangostin (12.5 and 25 mg/kg) also attenuated MDA, an indicator of lipid peroxidation, levels. Moreover, α-mangostin (12.5 and 25 mg/kg) alleviated the increased inflammatory cytokine TNFα, IL-1β, and IL-6 levels from the LPS/D-GalN treatment. α-Mangostin (12.5 and 25 mg/kg) also significantly decreased the amount of cell necrosis and inflammatory cell infiltration from the LPS/D-GalN treatment. Another in vivo study by Fu et al. (2018), performed on acetaminophen (APAP)-induced acute liver injury in mice, showed the protective effects of α-mangostin (12.5 and 25 mg/kg) [54]. A 6-day pretreatment with α-mangostin decreased the serum levels of AST and ALT induced by APAP. α-Mangostin (12.5 and 25 mg/kg) attenuated hepatic oxidative stress by increasing antioxidant enzyme activity, shown by decreasing MDA levels and increasing SOD activity and GSH. α-Mangostin (12.5 and 25 mg/kg) also showed anti-inflammatory effects through alleviated cell necrosis, the inhibition of elevated APAP-induced IL-1β, IL-6, and TNFα levels, and significant dose-dependent decreases in the mRNA expression of IL-1β, IL-6, and TNFα. The α-mangostin pretreatment (12.5 and 25 mg/kg) also inhibited iNOS expression. One more in vivo study by Shehata et al. (2022) explored the protective effects of α-mangostin against liver injury induced by concanavalin A (Con A) in mice [55]. Con A injection caused significant liver damage, evident from elevated liver toxicity markers (ALT, ALP, LDH, AST, and γ-GT) and decreased albumin levels, alongside histopathological changes, including inflammatory cell infiltration and hydropic degeneration. α-Mangostin (25 and 50 mg/kg) pretreatment reduced these toxicity markers, improved albumin levels, and ameliorated liver histopathology. Con A also increased oxidative stress markers (MDA, PC, and 4-HNE) while reducing antioxidants (TAC, GSH, and SOD). α-Mangostin (25 and 50 mg/kg) pretreatment alleviated oxidative stress by decreasing lipid peroxidation and enhancing antioxidant levels. Additionally, Con A exposure raised the levels of pro-inflammatory markers (IL-6, NF-κB, and TNFα). α-Mangostin (25 and 50 mg/kg) pretreatment significantly lowered these pro-inflammatory markers, demonstrating its anti-inflammatory and hepatoprotective potential against Con A-induced liver injury [55].

### 3.4. Neurological Disorders

The anti-neuroinflammatory benefits of α-mangostin have also been demonstrated in various studies. In an in vivo study, Catorce and colleagues (2016) expanded on the potent anti-neuroinflammatory effects of α-mangostin in LPS-induced C57BL/6J mice [56]. ELISA analyses proved α-mangostin (40 mg/kg) treatment to be successful in attenuating the LPS-stimulated levels of the pro-inflammatory marker IL-6 in the brain, whereas those of IL-1β and TNFα remained unaltered. Additionally, the background and LPS-induced brain tissue protein expression of COX-2, an enzyme associated with inflammation and the synthesis of prostaglandins [57], was also significantly downregulated by treating mice with α-mangostin (40 mg/kg). Moreover, it has been established that an inflammatory response may be detected by the upregulation of 18 kDa translocator protein (TSPO) expression [58]. To ascertain if α-mangostin could reduce TSPO expression, Catorce et al. (2016) employed a monoclonal anti-TSPO antibody to measure the amount of TSPO in the brains of rats. When comparing the hippocampal and cortical regions of the LPS-induced rats to control animals, immunofluorescence labeling revealed elevated TSPO expression. Interestingly, mice fed with α-mangostin (40 mg/kg) prior to LPS induction showed a significant decrease in TSPO immunoreactivity. Lastly, the hippocampal and cortical regions of the LPS-treated rats also showed an elevation of Iba-1 immunoreactivity, a frequently used marker for activated microglia. However, the α-mangostin (40 mg/kg)-treated mice showed a marked reduction in Iba-1 protein levels in the hippocampus but not in the cortex. This was the first study to prove the anti-neuroinflammatory potential of α-mangostin in an LPS-induced animal model, suggesting that the xanthone mainly reduces brain endothelial cell activation, since TSPO and COX-2 co-localize with the endothelial cell marker CD31 but not with astrocytes or microglia [56]. Researchers have also examined the potential of α-mangostin to exert its anti-inflammatory effects when associated with psychiatric disorders. In an in vivo study, Lotter et al. (2020) investigated the effects of α-mangostin and raw *G. mangostana* pericarp versus an antipsychotic drug, haloperidol, on a maternally induced immune-inflammatory model of schizophrenia in male Sprague Dawley rats [59]. Their results found that the LPS-stimulated plasma levels of IL-6 were downregulated by both α-mangostin (20 mg/kg) and the raw fruit treatment groups after 16 days, while only raw *G. mangostana* pericarp downregulated TNFα plasma levels. They concluded that, although both α-mangostin and the raw fruit proved to have strong antidepressant-like and anti-inflammatory qualities while also enhancing the haloperidol-induced anti-immobility reaction, further dose–response and clinical investigations are needed to confirm whether they can provide therapeutic advantages [59]. In another combined in vitro and in vivo study, α-mangostin was found to inhibit microglial inflammation causing memory impairment by suppressing the TAK1 and NF-κB signaling pathways [60]. α-Mangostin (50–1000 nM) dose-dependently inhibited protein levels of TNFα, IL-6, and iNOS, as well as nitric oxide production, in LPS-stimulated BV-2 microglial cells, with the highest inhibition rate at 500 nM. A pretreatment of α-mangostin (500 nM) for 1 h followed by LPS stimulation for 24 h showed the suppression of microglial migration towards a scratch on a wound-healing assay. α-Mangostin (500 nM) pretreatment also resulted in a decrease in the number of phagocytic cells and events in the LPS-stimulated BV-2 microglial cells. Additionally, α-mangostin (500 nM) reversed LPS-induced neuronal death and dendritic loss in mouse hippocampal neurons. In vivo, α-mangostin treatment (50 mg/kg) also suppressed LPS-induced dendritic damage, as shown by a stronger MAP2 reactivity. Moreover, in the hippocampus and cerebral cortex of LPS-treated mice, Western blot analysis showed increased iNOS and ELISA showed increased TNFα and IL-6, which were all repressed by α-mangostin. Another in vitro study by Hu et al. (2016) investigated the neuroprotective effects of α-mangostin in α-synuclein-induced neuroinflammation and neurotoxicity, common features in neurodegenerative diseases [61]. α-synuclein activation in rat primary microglial cells increased the production of pro-inflammatory cytokines (IL-1β, IL-6, TNFα), NO, and ROS. α-Mangostin (1, 10, and 100 nM) significantly reduced the cytokine and NO production in a dose-dependent manner. α-Mangostin (1, 10, and 100 nM) also inhibited α-synuclein-induced ROS production, targeting NADPH oxidase (NOX-1) activity. In neuron–microglia co-cultures, α-mangostin (1, 10, and 100 nM) protected dopaminergic (DA) neurons from microglia-mediated neurotoxicity by reducing α-synuclein-induced microglial activation. DA neuron survival assays showed that α-mangostin (1, 10, and 100 nM) directly promoted DA neuron survival, particularly at 10 nM. In neuron–microglia co-cultures, α-mangostin (1, 10, and 100 nM) treatment after α-synuclein exposure reversed the reduction in neuronal viability, demonstrating α-mangostin’s protective effects through the modulation of microglial activation and direct neuroprotection [61].

### 3.5. Respiratory Disorders

The anti-inflammatory benefits of α-mangostin may also have therapeutic effects in respiratory issues. For example, in a combined in vitro and in vivo study, the activation of the cholinergic anti-inflammatory pathway (CAP) through the α-mangostin treatment of LPS-induced acute lung injury (ALI) was explored in male Sprague Dawley rats and RAW 264.7 cells [62]. ALI perpetuated a cytokine storm both in vivo and in vitro, both of which were ameliorated by α-mangostin treatment. α-Mangostin (40 mg/kg) reduced the LPS-induced serum levels of IL-β and TNFα in vivo, and α-mangostin (5 μg/mL) suppressed the production of these cytokines in RAW 264.7 macrophages in vitro. Additionally, α-mangostin (40 mg/kg) sustained the activation of the CAP in vivo through the increase in the peripheral amounts of ACh and α7nAchR expression, while α-mangostin (5 μg/mL) produced similar effects in vitro, shedding light on its therapeutic potential for cholinergic-associated diseases [62]. In another combined in vitro and in vivo study, α-mangostin was tested for its potential in alleviating LPS-induced ALI in male Sprague Dawley rats and in the RAW 264.7 cell line [63]. α-Mangostin (15 and 45 mg/kg) markedly decreased the LPS-induced levels of interalveolar septal thickening, alveolar hemorrhage, and inflammatory cell infiltration, as well as the leucocyte populations of monocytes and neutrophils [63], as was also observed in the study by Yang and colleagues (2020) [62]. Similarly, dose-dependent α-mangostin treatment significantly inhibited the overproduced serum levels of TNFα while reversing the suppression in SOD activity [63]. Another in vivo study by Jang et al. (2012) examined the effects of α-mangostin in mitigating ovalbumin (OVA)-induced allergic asthma in BALB/c mice [64]. After a three-day treatment period, α-mangostin (10 and 30 mg/kg) significantly and dose-dependently decreased OVA-induced eosinophil and neutrophil cell infiltration in the lungs and bronchoalveolar lavage fluid (BALF), suppressed levels of peribronchial fibrosis, and significantly reduced Penh values, indicative of suppressed airway hyper-responsiveness [64]. Additionally, immunoassays demonstrated that α-mangostin therapy significantly reduced elevated BALF levels of TGF-1β, IL-4, IL-5, and IL-13, as well as total and OVA-specific IgE serum and BALF levels [64]. The collective results of these studies support the role of α-mangostin in ameliorating respiratory disorders, including acute lung injury and asthma.

Table 1 highlights the main signaling mediators responsible for the reported anti-inflammatory and immunomodulatory effects of α-mangostin in different disorders.

## 4. Immunomodulatory Effects of α-Mangostin

In addition to the anti-inflammatory properties of α-mangostin, discussed above, α-mangostin has also been investigated for its immunomodulatory properties, including innate immunity, the body’s first line of defense, and adaptive immunity, specific responses mediated by B and T lymphocytes. These involve both cellular and humoral immune responses. Understanding the effects that α-mangostin has on different types of immunity is necessary for determining its therapeutic potential in various conditions and disorders.

### 4.1. Effects on T Cells and Adaptive Immunity

α-Mangostin has been shown to influence adaptive immunity by modulating T cell activity, particularly in conditions associated with excessive immune responses. Shehata et al. (2022) demonstrated that α-mangostin (25 and 50 mg/kg) reduced CD4^+^ T cell infiltration in a concanavalin A (Con A)-induced liver injury model, suggesting a hepatoprotective role [55]. Further studies on T cell regulation have highlighted α-mangostin’s potential role in autoimmune diseases. In an experimental collagen-induced arthritis (CIA) model, α-mangostin (40 mg/kg) reduced the levels of anti-CII IgG2a antibodies, which play a crucial role in rheumatoid arthritis (RA) pathogenesis [39]. In vitro, α-mangostin (5 μg/mL) exhibited cytotoxicity against Th1 and Th17 polarized lymphocytes, decreasing their viability by 37% [39]. Given that Th1 and Th17 cells mediate inflammatory and autoimmune responses, these findings suggest a role for α-mangostin in immune modulation by limiting T cell activation and cytokine production. Furthermore, α-mangostin treatment has been linked to altered T cell differentiation. In a study by Yin et al. (2020), α-mangostin (5 μg/mL) was found to significantly suppress Th17 populations while decreasing IL-1β and TNFα production [40]. Additional studies have shown that α-mangostin inhibits IFNγ-positive cells and restores the Th1/Treg cell ratio [33], further supporting its immunomodulatory potential.

### 4.2. Effects on B Cells and Humoral Immunity

Beyond its role in T cell modulation, α-mangostin has been implicated in regulating humoral immunity. Jang et al. (2012) demonstrated that α-mangostin (10 and 30 mg/kg) significantly reduced BALF levels of IL-4, IL-5, IL-13, and TGF-1β in an ovalbumin (OVA)-induced allergic asthma model in BALB/c mice [64]. The reduction in IL-4 and IL-13 suggests that α-mangostin suppresses B cell differentiation and IgE secretion, inhibiting Th2-mediated immune responses. These findings indicate that α-mangostin plays a role in modulating antibody production and allergic responses, further broadening its immunomodulatory applications.

### 4.3. Effects on Macrophages and Innate Immunity

α-Mangostin also influences innate immune responses, particularly macrophage polarization and migration. In LPS-stimulated murine bone marrow-derived dendritic cells (mBMDCs), α-mangostin (7 and 10 μg/mL) significantly downregulated the percentage and mean fluorescent intensity (MFI) of CD86 and CD40, reducing antigen-presenting cell activation [39]. Additionally, α-mangostin (3 and 5 μg/mL) decreased IL-12 levels while increasing IL-10 production, reinforcing its anti-inflammatory effects [39]. Macrophage migration and polarization are also affected by α-mangostin. Li et al. (2019) found that α-mangostin (25 and 50 mg/kg) suppressed the expression of MCP-1, Mip-1α, and the chemokines CXCL10, CCL11, CX3CL1, and CCL5, reducing macrophage infiltration in adipose tissue [46]. Furthermore, α-mangostin treatment induced an anti-inflammatory M2 macrophage shift by decreasing M1 markers (CD11c) and increasing M2 markers (CD206 and Arg-1) [46]. Similarly, Kim et al. (2017) observed that α-mangostin (50 mg/kg/day) decreased M1 markers while promoting an M2 phenotype, suggesting a role in mitigating chronic inflammation [51]. In vitro, α-mangostin (10–80 μM) enhanced macrophage phagocytosis while promoting an M2 shift in LPS-stimulated macrophages [28]. Collectively, these findings highlight α-mangostin’s capacity to modulate macrophage activity, reducing inflammation and enhancing immune homeostasis.

## 5. Signaling Pathways Underlying Immunomodulatory and Anti-Inflammatory Effects of α-Mangostin

Numerous studies have been conducted to uncover the underlying molecular pathways that are modulated by α-mangostin to provide its anti-inflammatory and immunomodulatory effects. The pathway most extensively studied for its modulation by α-mangostin has been the NF-κB pathway, followed by several links to the MAPK pathway. Other pathways involved include the Nrf2 and OSM pathways.

### 5.1. NF-κB Signaling Pathway

The NF-κB signaling pathway has been extensively studied in relation to the anti-inflammatory effects of α-mangostin. This pathway, typically activated by pro-inflammatory cytokines such as IL-1 and TNFα, regulates the expression of various pro-inflammatory genes. One study demonstrated that α-mangostin (3, 6, and 12 μM) pretreatment significantly suppressed p-p65, p-IκB, and p65 nuclear translocation, thereby inhibiting NF-κB activation in IL-1β-induced rat chondrocytes [36]. Tarasuk et al. (2022) similarly observed NF-κB inhibition in infected HepG2 cells treated with α-mangostin (5, 10, and 20 μM) [26]. Furthermore, in DENV-2-infected cells, α-mangostin (20 μM) downregulated cytokine transcription, reducing RANTES to 13%, IP-10 to 5%, TNFα to 5%, and IL-6 to 6% [26,27]. Zou et al. (2019) found that α-mangostin (2.5, 5, and 10 μM) suppressed the LPS-induced phosphorylation of the NF-κB upstream proteins p-TAK1 and p-IKK in IEC-6 cells [22].

Guan et al. (2020) further confirmed NF-κB inhibition in LPS-stimulated BV-2 microglial cells with α-mangostin (500 nM), reducing the phosphorylation and nuclear translocation of p65 [60]. This effect was also observed in vivo, where α-mangostin (50 mg/kg) reduced LPS-induced p65 and TAK1 phosphorylation in C57BL/6 mice. Tao et al. (2018) linked α-mangostin’s effects to TLR4/NF-κB suppression in RAW 264.7 macrophages, significantly downregulating TNFα, TLR4, and HMGB [63]. Additional studies reported reductions in p65 phosphorylation and nuclear translocation upon α-mangostin treatment (5–12 μg/mL) in different inflammatory models [35,62]

Jang et al. (2012) found that α-mangostin (10 and 30 mg/kg) attenuated NF-κB activation in OVA-induced allergic asthma in BALB/c mice [64]. Other studies further demonstrated NF-κB inhibition in liver injury models, correlating it with increased Nrf2 and HO-1 expression, indicating antioxidant effects [53,55]. α-Mangostin (12.5 and 25 mg/kg) was also found to suppress APAP-induced IκBα degradation and NF-κB p65 translocation [54].

### 5.2. MAPK Signaling Pathway

The MAPK pathway is another major target of α-mangostin’s anti-inflammatory effects. This pathway consists of protein kinases involved in phosphorylation cascades leading to extracellular signal-regulated kinase (ERK) translocation and the activation of transcription factors [65]. Bumrungpert et al. (2010) found that α-mangostin (3, 10, and 30 μM) suppressed the LPS-induced phosphorylation of JNK, p38, and ERK in human adipocytes and macrophages [50]. Similarly, α-mangostin inhibited AP-1 activity by suppressing c-Jun phosphorylation, a downstream target of JNK [49].

Im et al. (2017) highlighted the photoprotective effects of α-mangostin (100 mg/kg) through its inhibition of MAPK phosphorylation in UVB-exposed HR-1 mice [24]. Li et al. (2019) demonstrated that α-mangostin (10–50 mg/kg) reversed LPS-suppressed SIRT3 expression, thereby suppressing the MAPK and NF-κB pathways in the eWAT of young and old mice. Further support for this mechanism was found when knocking down SIRT3 prevented the anti-inflammatory effects of α-mangostin in RAW 264.7 macrophages [46].

Liu et al. (2012) investigated α-mangostin’s effects on LPS-stimulated U937 cells, where α-mangostin (7.6–30.5 nM) dose-dependently suppressed inflammatory cytokines TNFα and IL-4. Gene expression analysis revealed the downregulation of 183 inflammation-related genes, including *IRAK2*, *NF-κB*, and *IL-6*. MAPK phosphorylation was inhibited, with α-mangostin (6–12 nM) reducing p38 phosphorylation to 38% of LPS-stimulated levels and downregulating STAT1, c-Jun, and c-Fos [66].

### 5.3. Nrf2 Signaling Pathway and Antioxidant Effects

The Nrf2 signaling pathway plays a key role in oxidative stress responses, with α-mangostin demonstrating significant modulation. Shehata et al. (2022) showed that α-mangostin (25 and 50 mg/kg) upregulated protective genes such as *SIRT1*, *Nrf2*, *NQO1*, *HO-1*, and *GCL* in a Con A-induced liver injury model [55]. Another study further confirmed the antioxidant role of α-mangostin in LPS/D-GalN-induced inflammation, where it increased Nrf2 and HO-1 levels [53]. Shen et al. (2014) identified α-mangostin’s inhibition of *Nrf2* expression in 3T3-L1 adipocytes, suppressing NF-κB activation during adipogenesis and preventing inflammation [67].

### 5.4. Oncostatin M (OSM) Signaling Pathway

The OSM signaling pathway, involved in autoimmune diseases such as rheumatoid arthritis and multiple sclerosis, has also been implicated in α-mangostin’s anti-inflammatory effects. Liu et al. (2012) found that α-mangostin modulated OSM signaling in LPS-stimulated U937 cells, reducing inflammation-related gene expression and suppressing MAPK activity [66].

### 5.5. Crosstalk Between Signaling Pathways

Evidence suggests that α-mangostin mediates its immunomodulatory and anti-inflammatory effects through interactions between multiple pathways. For example, the inhibition of NF-κB is often accompanied by MAPK suppression [46,66]. Additionally, the interplay between the NF-κB and Nrf2 pathways may contribute to α-mangostin’s dual anti-inflammatory and antioxidant effects [53,55]. These findings highlight the need for further research into how α-mangostin influences less-studied pathways such as the OSM signaling pathway. Overall, α-mangostin exerts potent anti-inflammatory effects through the NF-κB, MAPK, Nrf2, and OSM pathways. While NF-κB and MAPK remain its primary targets, additional research is needed to fully elucidate the extent of α-mangostin’s effects on minor immune pathways.

Table 2 provides a detailed summary of the main findings related to the anti-inflammatory and immunomodulatory effects of α-mangostin.

## 6. Conclusions

α-Mangostin has emerged as a promising natural compound with potent anti-inflammatory and immunomodulatory effects. Through extensive in vitro and in vivo studies, α-mangostin has been shown to modulate key inflammatory pathways, including NF-κB, MAPK, and Nrf2, thereby reducing inflammation and oxidative stress. α-Mangostin demonstrates promising anti-inflammatory activity in preclinical studies, placing it mechanistically between NSAIDs and biologics. Unlike NSAIDs, which are associated with gastrointestinal and renal toxicity, α-mangostin exhibits a more favorable safety profile in animal models. Compared to biologics, it poses a lower risk of immunosuppression and is significantly more cost-effective. Additionally, its immunomodulatory capabilities extend to influencing macrophage polarization, T cell responses, and cytokine production, making it a potential candidate for treating a range of inflammatory and immune-related disorders. Despite these promising findings, further research is necessary to elucidate its precise mechanisms, optimize its bioavailability, and assess its long-term safety in clinical settings. Future investigations should focus on translating these preclinical findings into clinical applications to harness the full therapeutic potential of α-mangostin as a natural anti-inflammatory and immunomodulatory agent.

## Figures and Tables

**Table 1:** The signaling mediators responsible for the reported anti-inflammatory and immunomodulatory effects of α-mangostin in different disorders. The adjacent arrows next to a molecule indicate a decrease in that particular molecule when pointed down or an increase when pointed up, and are based on the general trend of the effects for each disorder.

Joint Disorders	Digestive and Metabolic Disorders	Hepatic Disorders	Neurological Disorders
↓ paw swelling	↓ ROS	↓ macrophage infiltration	↓ IL-6
↓ joint deformation	↓ NO	↓ MCP-1	↓ COX-2
↓ granulocytes	↓ MPO	↓ IL-6	↓ TSPO
↓ lymphocytes	↑ MCP-1	↓ AST	↓ Iba-1
↓ intermediate cells	↓ TLR-2	↓ ALT	↓ TNFα
↓ neutrophil cells	↓ TNFα	↓ TGF-β1	↓ iNOS
↓ platelet cells	↑ ICAM-1	↓ α-SMA	↓ NO
↓ IL-6	↓ VCAM-1	↓ TIMP-1	↓ microglial migration
↓ TGF-β	↑ colon length	↓ MDA	↓ phagocytic cells
↓ HIF-1α	↓ surface epithelial cell damage	↓ TNFα	↓ neuronal death
↓ VEGF	↓ inflammatory cell infiltration	↓ IL-1β	↓ dendritic damage
↓ COX-2	↓ submucosal edema	↓ cell necrosis	↓ ROS
↓ HUVEC migration	↓ IL-6	↑ antioxidant enzymes	↓ IL-1β
↓ TNFα	↓ IL-1β	↑ SOD	↑ neuron survival
↓ IL-β	↓ COX-2	↓ iNOS	
↑ hemoglobin count	↓ iNOS	↓ ALP	**Respiratory Disorders**
↑ NAMPT	↓ triglyceride levels	↓ LDH	↓ IL-1β
↑ SIRT1	↓ LDL-C	↓ γ-GT	↓ TNFα
↑ PPARγ	↓ cholesterol	↓ lipid peroxidation	↑ α7nAChR
↑ glucose	↓ AST	↓ NF-κB	↑ Ach
↑ triglyceride levels	↓ ALT		↓ interalveolar septal thickening
↓ IL-1β	↓ miR-155		↓ alveolar hemorrhage
↑ SOD	↓ IL-8		↓ inflammatory cell infiltration
↓ MCP-1	↓ IFNg-IP-10		↓ leucocytes
↓ NAMPT	↑ PPARγ		↑ SOD
↓ NAD+			↓ peribronchial fibrosis
↓ synovial hyperplasia			↓ IL-4
↑ collagen II			↓ IL-5
↑ Aggrecan			↓ IL-13
↑ SOX-9			↓ IgE
↓ MMP-13			
↓ ADAMTS-5			
↓ matrix degradation			
↓ cartilage damage			
↓ PGE2 expression			
↓ NO expression			
↓ p-p65			
↓ p-IκB			
↓ iNOS			
↓ MMP-3			
↓ MMP-9			
↓ CXCL5			
↓ CXCL10			
↓ CCL5			
↓ CXCL9			
↓ IL-33			
↓ IL-12			
↓ IL-10			
↑ α7nAChR			
↑ ACh			

**Table 2:** A detailed summary of the main findings related to the anti-inflammatory and immunomodulatory effects of α-mangostin and its dosages, dosage regimens, and experimental models, as well as whether the reported effects were dose-dependent and/or time-dependent.

Main Effects	Experimental Model	Dosage	Administration Mode	Administration Duration	References
- Restoration in AIA-induced levels of granulocytes, lymphocytes, and intermediate cells - Suppression of AIA-induced paw swelling and joint deformation - Restoration in AIA- and LPS-induced IL-6 and TGF-β serum levels - Suppression of serum AIA-induced HIF-1α and LPS-induced COX-2 levels - Suppression of AIA- and LPS-induced VEGF levels in serum, synovium, and HUVECs	Male Wistar rats HUVEC cell line	30 mg/kg 2, 4, and 6 μg/mL	Oral administration	32 days, daily 24 h	[32]
- Increased articular cartilage thickness and amelioration of cartilage damage - Reduced expression of PGE_2_, NO, p-p65, and p-IκB - Inhibited release of iNOS, COX-2, MMP-3, MMP-9, and MMP-13 - Reduced activation of NF-κB p65 nuclear translocation - Increased expression of collagen II and Aggrecan	Male Sprague Dawley rats Rat chondrocytes	10 mg/kg 1.25, 2.5, and 5.0 μg/mL	Intraperitoneal administration	8 weeks, on alternate days 24 h	[38]
- Elevation in lymphocyte count - Restoration of expression of NAMPT in rat models - Restoration of AIA-elevated total triglyceride and glucose levels - Reversed decrease in SOD activity - Suppression of AIA-induced IL-1β and IL-6 serum levels - Restoration of AIA-induced SIRT1 and PPARγ levels in rat models and pre-adipocytes - Restoration of co-culture-induced elevation in IL-1β, IL-6, and MCP-1 levels in pre-adipocytes	Male Sprague Dawley rats Pre-adipocytes	50 mg/kg 4 μg/mL	Oral administration	30 days 24 h	[34]
- Reduction in histological scores of CIA-induced mononuclear cells and fibroblasts in mice - Suppression of IL-6, CXCL5, CXCL10, CXCL9, and CCL5 levels in mice - Downregulation of IL-33 expression in mice joint tissues - Reduction in CIA-induced levels of anti-CII IgG2a antibodies - Reduction in IL-12 production and elevation in IL-10 production in mBMDCs - Downregulation of CD86 and CD40 expression and MFI in mBMDCs	Male DBA/1J mice mBMDCs	40 mg/kg 1, 3, 5, 7, and 10 μg/mL	Oral administration	33 days, daily 4 h	[39]
- Reduction in LPS-induced serum, eWAT, and RAW macrophage levels of IL-6, TNFα, and MCP-1 - Suppression of LPS-elevated eWAT gene expression of F4/80, CD68, and M1 macrophage marker CD11c - Increased M2 markers, CD206, and Arg-1 - Reversal of LPS-induced iNOS in mice and in macrophages - Decreased LDL-C, triglycerides, cholesterol, AST, and ALT - Suppressed miR-155 in serum and eWAT - Reduction in LPS-induced serum and eWAT levels of IL-6, TNFα, MCP-1, IL-1β, and COX2 - Suppression of IKKα/β, IκBα, p65, ERK, and p38 phosphorylation in eWAT - Suppression of macrophage-related chemokines CXCL10, CCL11, CX3CL1, and CCL5 in mice, eWAT, and RAW cells - Suppression of miR-155 in RAW 264.7 cells - Suppression of IKK activation in RAW cells - Elevated LPS-suppressed SIRT3 expression in eWAT (all concentrations) and RAW cells	Male C57BL/6J mice RAW 264.7 cells	10 mg/kg 25 and 50 mg/kg 5 μM	Intraperitoneal administration Oral administration	5 days, daily 8 weeks, daily Pretreatment for 1 h	[46]
- Reduction in macrophage infiltration and inflammatory cytokines in liver and WAT - Decrease in CD11c M1 marker, TNFα, MCP-1, CCR2, and IL-6 - Suppression of migration ability of stimulated macrophages - Increase in CD206 M2 marker and IL-10	Male C57BL/6 mice	50 mg/kg/day	Oral administration	12 weeks	[51]
- Reduction in LPS-induced NO production - Raised TNFα secretion - Reduction in LPS-stimulated IL-8 release - Reduction in PMA-stimulated TNFα release of HepG2 cells - Reduction in LPS-stimulated IL-8 release of Caco-2 and HT-29 cells	RAW 264.7 macrophages MDM cells THP-1 cells HepG2 cells Caco-2 HTB-37 cells HT-29 cells	1, 3, and10 μM 4.5 μM 10 μM 7 μM 15 μM 10 μM	N/A N/A N/A N/A N/A N/A	Pretreatment for 2 h Pretreatment for 4 h Pretreatment for 4 h Pretreatment for 16 h Pretreatment for 4 h Pretreatment for 1 h	[20]
- Suppression of DSS-induced serum levels of ROS, NO, and MPO - Restoration of DSS-induced MCP-1 colonic mRNA expression - Restoration of DSS-induced colonic ICAM-1 - Downregulation of DSS-induced TLR-2 and TNFα mRNA expression - Downregulation of DSS-induced colonic VCAM-1	Male ICR mice	30 mg/kg	Oral administration	Pretreatment for 7 days, daily	[42]
- Downregulation of LPS-induced IL-6 and COX-2 cortical protein levels - Decrease in LPS-induced hippocampal and cortical TSPO immunoreactivity - Reduction in hippocampal Iba-1 protein levels	Female C57BL/6J mice	40 mg/kg	Oral administration	Pretreatment for 14 days, daily	[56]
- Downregulation of LPS-induced plasma levels of IL-6 - Increase in haloperidol-induced anti-immobility reaction	Male Sprague Dawley rats	20 mg/kg	Oral administration	16 days, daily	[59]
- Suppression of LPS-induced production of NO, PGE2, IL-6, TNFα, and IL-1β - Suppression of LPS-induced mRNA expression of iNOS, COX-2, IL-6, TNFα, IL-1β, TLR4, and MYD88 - Downregulation of p-TAK1 and p-IKK, and nuclear import of p65, p-IκB, and p-p65 expression - Upregulation of IκB expression	IEC-6 cells	2.5, 5, and 10 μM	N/A	Pretreatment for 1 h	[22]
- Suppression of circulating TNFα, IFNγ, IL-1β, ALT, and AST after CLP induction - Upregulation of IL-10 in CLP-induced mice - Suppression of LPS-induced mRNA and protein levels of NLRP3, ASC, Caspase1, and IL-1β - Increase in macrophage phagocytosis and M2-type macrophage shift	Male BALB/c mice Murine macrophages	3 mg/kg 10, 20, 40, 80 μM	Intravenous administration	7 days 24 h	[28]
- Reduction in TAA-induced AST and ALT hepatic levels - Downregulation of TAA-induced TGF-β1, α-SMA, and TIMP-1	Male Wistar rats	50 mg/kg	Intraperitoneal administration	8 weeks, twice weekly	[52]
- Reduction in LPS-induced interalveolar septal thickening, alveolar hemorrhage, and inflammatory cell infiltration - Inhibition of serum TNFα - Reversal of LPS-induced suppression in SOD activity - Downregulation of LPS-induced levels of TNFα, TLR4, HMGB, and p-p65 in RAW 264.7 cells	Male Sprague Dawley rats RAW 264.7 macrophages	15 and 45 mg/kg 1, 3, and 5 μg/mL	Oral administration	3 days 4 h	[63]
- Suppression of LPS-induced mRNA expression of TNFα, IL-1β, IL-6, IL-8, MCP-1, and TLR-2 - Suppression of LPS-induced p-JNK, p-p38, and p-ERK - Suppression of transcriptional activity of AP-1 p-c-Jun	Human primary adipocytes	3 μM	N/A	24 h	[49]
- Suppression of IFNγ IP10 - Increase in LPS-suppressed PPARγ and adiponectin mRNA - Suppression of LPS-induced p-JNK, p-p38, and p-ERK	Human U937 monocytes	3, 10, and 30 μM	N/A	2 h	[50]
- Increase in peripheral amounts of ACh and α7nAchR expression in vitro and in vivo - Reduction in LPS-induced serum levels of IL-β and TNFα and their production in RAW 264.7 cells - Reduction in LPS-induced phosphorylation of p65 and nuclear aggregation of p65 in RAW 264.7 cells	Male Sprague Dawley rats RAW 264.7 cells	40 mg/kg 5 μg/mL	Oral administration N/A	3 days 0.5, 1, 2, 4, and 6 h	[62]
- Reduction in RA-induced inflammatory cell infiltration, articular cartilage erosion, and splenic lymphocyte density in rats - Restoration of RA-declined α7nAChR expression in rats - Downregulation of circulating levels of IL-β and TNFα in rats and human PBMCs - Reduction in AChE catalytic activity in rat PBMCs - Suppression of LPS-stimulated Th17 populations in human PBMCs - Decrease in Th1 and Th2 populations in human PBMCs	Male Sprague Dawley rats Rat and human PBMCs	40 mg/kg 2.5, 5, and 10 μg/mL	Oral administration	45 days 2 h	[40]
- Restoration of RA-declined α7nAChR expression in rats - Decrease in Th1 populations in human PBMCs	Male Sprague Dawley Rats RAW 264.7 macrophages	40 mg/kg 5 μg/mL	Oral administration	45 days 24 h	[41]
- Inhibition of synovium expression of NAMPT and NAD^+^ in serum - Suppression of NAMPT and NAD - Reduced LPS-induced elevation of TNFα and p-p65	Male Sprague Dawley rats Human FLSs	40 mg/kg 8, 10, and 12 μg/mL	Oral administration	45 days 6 h	[35]
- Reduction in OVA-induced eosinophil and neutrophil cell infiltration in lungs and BALF - Reduction in OVA-elevated BALF levels of TGF-1β, IL-4, IL-5, and IL-13 - Reduction in OVA-elevated IgE serum and BALF levels - Suppression of OVA-induced lung tissue levels of nuclear p65 - Suppression of OVA-induced lung tissue levels of phosphorylated Akt and PIP3	Female BALB/c mice	10 and 30 mg/kg	Oral administration	3 days	[64]
- Downregulation of CXCL17 and IL-23α levels in rats - Downregulation of LPS-elevated mRNA and protein expression of NLRP3, caspase 1, IL-1β, and IL-18	Male Sprague Dawley rats IEC-6 cells	50 mg/kg 10 μM	Intragastrical administration	Pretreatment for 5 days, every day Pretreatment for 1 h	[23]
- Inhibition of UVB-induced wrinkle formation - 2-fold increase in epidermal thickness - Suppression of UVB-induced mRNA expression of IL-1β, IL-6, and TNFα, and protein expression of MMP-1 and -9 - Increase in SOD catalytic activity - Suppression of p-ERK, p-p38, and p-JNK	Male HR-1 mice (UVB-induced inflammation)	100 mg/kg	Oral administration	Pretreatment for 12 weeks	[24]
- Inhibitory effect on NO release with IC_50_ value of 3.1 μM for α-mangostin - Inhibitory effect on PGE2 release with IC_50_ value of 13.9 μM for α-mangostin - Moderate effects on TNFα and IL-4 releases with IC_50_ values 31.8–64.8 μM for α- and γ-mangostin - Suppressed gene transcription for iNOS and inhibited COX-2 expression	RAW 264.7 macrophage cells	3–100 μg/mL 3, 10, and 30 μM	N/A	Incubated for 48 h Incubated for 20 h	[21]
- Dose-dependent inhibition of DENV infection - Suppressed transcription of RANTES, IP-10, TNFα, and IL-6 in DENV-1-, DENV-2-, DENV-3-, and DENV-4-infected cells	Huh-7 cells	10, 15, and 20 μM	N/A	24, 48, and 72 h	[27]
- Suppressed transcription of RANTES, IP-10, TNFα, and IL-6 in DENV-2-infected cells - Dose-dependent inhibition of DENV infection - Downregulation of all DENV-2 protein expression - Inhibited DENV life cycle by reducing viral replication - Inhibited NF-κB nuclear translocation - Inhibited DENV-2 RdRp activity, IC_50_ = 16.50 μM	HepG2 cells	5, 10, 20, and 40 μM	N/A	24 and 48 h	[26]
- Viral reduction by more than 50% - Reduced TNFα and IFNγ expression	Human peripheral blood mononuclear cells	5, 10, and 20 μM	N/A	24 and 48 h	[25]
- Suppressed PPARγ expression by 40% - Dose-dependent inhibition of Nrf2	NF-κB-RE/GFP adipocytes Nrf2-P/YFP adipocytes	10 μM 1–20 μM	N/A	7 days 8 days	[67]
- Reduced inflammatory leucocytes, neutrophils, lymphocytes, and platelet cells - Reduced expression of inflammatory cytokines TNFα and IL-β - Decreased IFNγ positive cells and increased FOXP3 expression restoring Th1/Treg cell ratio - Abolished increased effects of p-p65 and decreased VEGF (downstream target of NF-κB) expression) - Inhibited HFLS-RA cell proliferation at high concentrations (above 8 μg/mL) - Activated NF-κB at lower concentrations while inhibiting NF-κB activation at higher concentrations and longer treatment times	Male Sprague Dawley rats HFLS-RA cells	40 mg/kg 6, 8, 10, 12, and 14 μg/mL	Intragastric administration N/A	35 days, daily 24 h	[33]
- Dose-dependent inhibition of IL-1β-induced degradation of collagen II and Aggrecan degradation - Reverted IL-1β-induced expression of p-p65 and p-IκB, decrease in SOX-9, and release of MMP-13 and ADAMTS-5 - Dose-dependent reversion of increased cell apoptosis and decreased cell viability induced by IL-1β - Increase in thickness of articular cartilage and reduced cartilage deterioration	Rat chondrocyte cells 6-week-old male Sprague Dawley rats	3, 6, and 12 μM 10 mg/kg	N/A Intraperitoneal injection	2 h Every other day for 8 weeks after OA-inducing surgery	[36]
- Dose-dependent inhibition of TNFα, IL-6, and iNOS, as well as nitric oxide production - Suppression of microglial migration - Decrease in number of phagocytic cells and events - Inhibited NF-κB pathway by decreasing phosphorylation and nuclear translocation of p65 - No effect on MAPK signaling pathway - Reversed LPS-induced neuronal death and dendritic loss - Suppressed LPS-induced dendritic damage, as shown by stronger MAP2 reactivity - Repression of LPS-induced iNOS, TNFα, and IL-6 - Decreased LPS-induced p65 and TAK1 phosphorylation, and TLR4 and MyD88 expression	BV-2 microglial cells Mouse hippocampal neurons Male C57BL/6 mice (20–25 g)	50–1000 nM 500 nM 100–500 nM 50 mg/kg	N/A Daily oral gavage	24 h Pretreatment for 1 h Pretreatment for 1 h Once per day for 14 days	[60]
- Significantly decreased AST and ALT levels - Attenuated MDA levels - Alleviated increased inflammatory cytokine TNFα, IL-1β, and IL-6 levels - Decreased the amount of cell necrosis and inflammatory cell infiltration - Inhibited LPS/D-GalN-induced increases in TLR4, phosphorylated NF-κB p65, and phosphorylated IκB - Inhibited LPS/D-GalN-induced increases in TLR4, phosphorylated NF-κB p65, and phosphorylated IκB - Increased Nrf2 and HO-1, indicating antioxidant effects	Adult male ICR mice (20–22 g)	12.5 and 25 mg/kg	Intragastric administration	Once daily for 7 days	[53]
- Decreased serum levels of AST and ALT induced by APAP - Attenuated hepatic oxidative stress by decreasing MDA levels and increasing SOD activity and GSH - Alleviated cell necrosis - Inhibition of elevated APAP-induced TNFα, IL-1β and IL-6 levels - Dose-dependent decrease in mRNA expression of IL-6, IL-1β, and TNFα - Inhibited iNOS expression - Inhibited NF-κB pathway by dose-dependently suppressing APAP-induced degradation of IκBα levels and translocation of NF-κBp65 - MAPK pathway inhibited by suppressing phosphorylation of ERK, JNK, and p38	4–5-week-old male ICR mice (20–25 g)	12.5 and 25 mg/kg	Intragastric administration	Once daily for 6 days	[54]
- Lowered disease activity index (DAI) scores - Longer colon lengths than DSS group - Reversal of myeloperoxidase (MPO) increase in esophagi and colons, indicating inhibition of leukocytes - Decreased DSS-induced upregulation of IKK and IκBα phosphorylation as well as activated NF-κB - Attenuated surface epithelial cell damage, inflammatory cell infiltration, and submucosal edema induced by DSS	6-week-old mice	30 and 100 mg/kg	Orally	3 days pretreatment followed by 10-day treatment (after DSS administration)	[44]
- Reduced IL-1β, IL-6, TNFα, and NO production and inhibited NF-κB pathway activation by reducing IκBα degradation and p65 phosphorylation and nuclear translocation - Inhibited α-synuclein-induced ROS production, targeting NADPH oxidase (NOX-1) activity - Protected dopaminergic (DA) neurons from microglia-mediated neurotoxicity by reducing α-synuclein-induced microglial activation	Primary rat microglial cells and mesencephalic neuron–glia cultures	1, 10, and 100 nM	N/A	24 h	[61]
- Reduced ALT, ALP, LDH, AST, and γ-GT; improved albumin levels; and ameliorated liver histopathology - Alleviated oxidative stress by decreasing lipid peroxidation and enhancing antioxidants TAC, GSH, SOD - Upregulated *SIRT1*, *Nrf2*, *NQO1*, *HO-1*, and *GCL* genes and their pathways, boosting Nrf2 binding and HO-1 levels - Lowered IL-6, NF-κB, and TNFα and reduced CD4^+^ T cell infiltration	Adult male Swiss albino mice (20–25 g)	25 and 50 mg/kg	Oral administration	7 days	[55]
- Inhibited TNFα and IL-4 - Altered pathways involved in immune responses, apoptosis, and cell death regulation, notably suppressing 183 genes (e.g., *IRAK2*, *NF-κB*, *OLR1*, *IL-6*, *CCR7*, *ORM1*, etc.) linked to inflammation - Affected OSM pathway and modulated MAPK signaling pathways, reducing phosphorylation of p38, ERK1/2, and JNK - Reduced expression of STAT1, c-Jun, and c-Fos	U937 cells	6.0, 7.6, 12.0, 12.5, 13.4, and 30.5 nM	N/A	4 h	[66]

## Data Availability

No new data were created or analyzed in this study. Data sharing is not applicable to this article.

## References

[1] Abdulkhaleq L.A., Assi M.A., Abdullah R., Zamri-Saad M., Taufiq-Yap Y.H., Hezmee M.N.M. (2018). The Crucial Roles of Inflammatory Mediators in Inflammation: A Review. Vet. World.

[2] Chen L., Deng H., Cui H., Fang J., Zuo Z., Deng J., Li Y., Wang X., Zhao L. (2018). Inflammatory Responses and Inflammation-Associated Diseases in Organs. Oncotarget.

[3] Medzhitov R. (2008). Origin and Physiological Roles of Inflammation. Nature.

[4] Chavez-Dominguez R., Perez-Medina M., Aguilar-Cazares D., Galicia-Velasco M., Meneses-Flores M., Islas-Vazquez L., Camarena A., Lopez-Gonzalez J.S. (2021). Old and New Players of Inflammation and Their Relationship with Cancer Development. Front. Oncol..

[5] Majdalawieh A.F., Fayyad M.W. (2015). Immunomodulatory and Anti-Inflammatory Action of Nigella Sativa and Thymoquinone: A Comprehensive Review. Int. Immunopharmacol..

[6] Majdalawieh A.F., Yousef S.M., Abu-Yousef I.A., Nasrallah G.K. (2022). Immunomodulatory and Anti-Inflammatory Effects of Sesamin: Mechanisms of Action and Future Directions. Crit. Rev. Food Sci. Nutr..

[7] Smith W.L., Murphy R.C. (2016). The Eicosanoids. Biochemistry of Lipids, Lipoproteins and Membranes.

[8] Ghlichloo I., Gerriets V. (2025). Nonsteroidal Anti-Inflammatory Drugs (NSAIDs).

[9] Majdalawieh A.F., Yousef S.M., Abu-Yousef I.A., Nasrallah G.K. (2022). Immunomodulatory and Anti-Inflammatory Effects of Berberine in Lung Tissue and Its Potential Application in Prophylaxis and Treatment of COVID-19. Front. Biosci.-Landmark.

[10] Majdalawieh A.F., Ahari S.H., Yousef S.M., Nasrallah G.K. (2023). Sesamol: A Lignan in Sesame Seeds with Potent Anti-Inflammatory and Immunomodulatory Properties. Eur. J. Pharmacol..

[11] Posadino A.M., Giordo R., Ramli I., Zayed H., Nasrallah G.K., Wehbe Z., Eid A.H., Gürer E.S., Kennedy J.F., Aldahish A.A. (2023). An Updated Overview of Cyanidins for Chemoprevention and Cancer Therapy. Biomed. Pharmacother..

[12] Majdalawieh A.F., Mansour Z.R. (2019). Sesamol, a Major Lignan in Sesame Seeds (*Sesamum Indicum*): Anti-Cancer Properties and Mechanisms of Action. Eur. J. Pharmacol..

[13] Alam M., Rashid S., Fatima K., Adnan M., Shafie A., Akhtar M.S., Ganie A.H., Eldin S.M., Islam A., Khan I. (2023). Biochemical Features and Therapeutic Potential of α-Mangostin: Mechanism of Action, Medicinal Values, and Health Benefits. Biomed. Pharmacother..

[14] Wittenauer J., Falk S., Schweiggert-Weisz U., Carle R. (2012). Characterisation and Quantification of Xanthones from the Aril and Pericarp of Mangosteens (*Garcinia Mangostana* L.) and a Mangosteen Containing Functional Beverage by HPLC–DAD–MSn. Food Chem..

[15] Watanapokasin R. (2011). Effects of α-Mangostin on Apoptosis Induction of Human Colon Cancer. World J. Gastroenterol..

[16] Jung H.-A., Su B.-N., Keller W.J., Mehta R.G., Kinghorn A.D. (2006). Antioxidant Xanthones from the Pericarp of *Garcinia Mangostana* (*Mangosteen*). J. Agric. Food Chem..

[17] Kim S.E., Yin M.Z., Roh J.W., Kim H.J., Choi S.W., Wainger B.J., Kim W.K., Kim S.J., Nam J.H. (2023). Multi-Target Modulation of Ion Channels Underlying the Analgesic Effects of α-Mangostin in Dorsal Root Ganglion Neurons. Phytomedicine.

[18] Majdalawieh A.F., Terro T.M., Ahari S.H., Abu-Yousef I.A. (2024). α-Mangostin: A Xanthone Derivative in Mangosteen with Potent Anti-Cancer Properties. Biomolecules.

[19] Kumar D. (2020). Molecular Biology of Acute and Chronic Inflammation. Clinical Molecular Medicine.

[20] Gutierrez-Orozco F., Chitchumroonchokchai C., Lesinski G.B., Suksamrarn S., Failla M.L. (2013). α-Mangostin: Anti-Inflammatory Activity and Metabolism by Human Cells. J. Agric. Food Chem..

[21] Tewtrakul S., Wattanapiromsakul C., Mahabusarakam W. (2009). Effects of Compounds from *Garcinia Mangostana* on Inflammatory Mediators in RAW 264.7 Macrophage Cells. J. Ethnopharmacol..

[22] Zou W., Yin P., Shi Y., Jin N., Gao Q., Li J., Liu F. (2019). A Novel Biological Role of α-Mangostin via TAK1–NF-κB Pathway against Inflammatory. Inflammation.

[23] Yin P., Zou W., Li J., Jin N., Gao Q., Liu F. (2019). Using High-Throughput Sequencing to Explore the Anti-Inflammatory Effects of α-Mangostin. Sci. Rep..

[24] Im A.-R., Kim Y.-M., Chin Y.-W., Chae S. (2017). Protective Effects of Compounds from *Garcinia Mangostana* L. (*Mangosteen*) against UVB Damage in HaCaT Cells and Hairless Mice. Int. J. Mol. Med..

[25] Sugiyanto Z., Yohan B., Hadisaputro S., Dharmana E., Suharti C., Winarto, Djamiatun K., Rahmi F.L., Sasmono R.T. (2019). Inhibitory Effect of Alpha-Mangostin to Dengue Virus Replication and Cytokines Expression in Human Peripheral Blood Mononuclear Cells. Nat. Prod. Bioprospect..

[26] Tarasuk M., Songprakhon P., Chieochansin T., Choomee K., Na-Bangchang K., Yenchitsomanus P. (2022). Alpha-Mangostin Inhibits Viral Replication and Suppresses Nuclear Factor Kappa B (NF-κB)-Mediated Inflammation in Dengue Virus Infection. Sci. Rep..

[27] Tarasuk M., Songprakhon P., Chimma P., Sratongno P., Na-Bangchang K., Yenchitsomanus P. (2017). Alpha-Mangostin Inhibits Both Dengue Virus Production and Cytokine/Chemokine Expression. Virus Res..

[28] Ge Y., Xu X., Liang Q., Xu Y., Huang M. (2019). α-Mangostin Suppresses NLRP3 Inflammasome Activation via Promoting Autophagy in LPS-Stimulated Murine Macrophages and Protects against CLP-Induced Sepsis in Mice. Inflamm. Res..

[29] Long H., Xu B., Luo Y., Luo K. (2016). Artemisinin Protects Mice against Burn Sepsis through Inhibiting NLRP3 Inflammasome Activation. Am. J. Emerg. Med..

[30] Jin L., Batra S., Jeyaseelan S. (2017). Deletion of Nlrp3 Augments Survival During Polymicrobial Sepsis by Decreasing Autophagy and Enhancing Phagocytosis. J. Immunol..

[31] Wu D., Shi L., Li P., Ni X., Zhang J., Zhu Q., Qi Y., Wang B. (2018). Intermedin1–53 Protects Cardiac Fibroblasts by Inhibiting NLRP3 Inflammasome Activation During Sepsis. Inflammation.

[32] Jiang T.-T., Ji C.-F., Cheng X.-P., Gu S.-F., Wang R., Li Y., Zuo J., Han J. (2021). α-Mangostin Alleviated HIF-1α-Mediated Angiogenesis in Rats with Adjuvant-Induced Arthritis by Suppressing Aerobic Glycolysis. Front. Pharmacol..

[33] Zuo J., Yin Q., Wang Y.-W., Li Y., Lu L.-M., Xiao Z.-G., Wang G.-D., Luan J.-J. (2018). Inhibition of NF-κB Pathway in Fibroblast-like Synoviocytes by α-Mangostin Implicated in Protective Effects on Joints in Rats Suffering from Adjuvant-Induced Arthritis. Int. Immunopharmacol..

[34] Hu Y.-H., Han J., Wang L., Shi C., Li Y., Olatunji O.J., Wang X., Zuo J. (2021). α-Mangostin Alleviated Inflammation in Rats with Adjuvant-Induced Arthritis by Disrupting Adipocytes-Mediated Metabolism-Immune Feedback. Front. Pharmacol..

[35] Yang K., Yin Q., Mao Q., Dai S., Wang L., Dong J., Zuo J. (2019). Metabolomics Analysis Reveals Therapeutic Effects of α-Mangostin on Collagen-Induced Arthritis in Rats by Down-Regulating Nicotinamide Phosphoribosyltransferase. Inflammation.

[36] Pan T., Chen R., Wu D., Cai N., Shi X., Li B., Pan J. (2017). Alpha-Mangostin Suppresses Interleukin-1β-Induced Apoptosis in Rat Chondrocytes by Inhibiting the NF-κB Signaling Pathway and Delays the Progression of Osteoarthritis in a Rat Model. Int. Immunopharmacol..

[37] Huang K., Wu L. (2008). Aggrecanase and Aggrecan Degradation in Osteoarthritis: A Review. J. Int. Med. Res..

[38] Pan T., Wu D., Cai N., Chen R., Shi X., Li B., Pan J. (2017). Alpha-Mangostin Protects Rat Articular Chondrocytes against IL-1β-Induced Inflammation and Slows the Progression of Osteoarthritis in a Rat Model. Int. Immunopharmacol..

[39] Herrera-Aco D.R., Medina-Campos O.N., Pedraza-Chaverri J., Sciutto-Conde E., Rosas-Salgado G., Fragoso-González G. (2019). Alpha-Mangostin: Anti-Inflammatory and Antioxidant Effects on Established Collagen-Induced Arthritis in DBA/1J Mice. Food Chem. Toxicol..

[40] Yin Q., Wu Y., Pan S., Wang D., Tao M., Pei W., Zuo J. (2020). Activation of Cholinergic Anti-Inflammatory Pathway in Peripheral Immune Cells Involved in Therapeutic Actions of α-Mangostin on Collagen-Induced Arthritis in Rats. Drug Des. Devel. Ther..

[41] Chen W.-G., Zhang S.-S., Pan S., Wang Z.-F., Xu J.-Y., Sheng X.-H., Yin Q., Wu Y.-J. (2022). α-Mangostin Treats Early-Stage Adjuvant-Induced Arthritis of Rat by Regulating the CAP-SIRT1 Pathway in Macrophages. Drug Des. Devel. Ther..

[42] Tatiya-aphiradee N., Chatuphonprasert W., Jarukamjorn K. (2021). Ethanolic *Garcinia Mangostana* Extract and α-Mangostin Improve Dextran Sulfate Sodium-Induced Ulcerative Colitis via the Suppression of Inflammatory and Oxidative Responses in ICR Mice. J. Ethnopharmacol..

[43] Kim J.J., Shajib M.S., Manocha M.M., Khan W.I. (2012). Investigating Intestinal Inflammation in DSS-Induced Model of IBD. J. Vis. Exp..

[44] You B.H., Chae H.-S., Song J., Ko H.W., Chin Y.-W., Choi Y.H. (2017). α-Mangostin Ameliorates Dextran Sulfate Sodium-Induced Colitis through Inhibition of NF-κB and MAPK Pathways. Int. Immunopharmacol..

[45] Gutierrez-Orozco F., Thomas-Ahner J.M., Berman-Booty L.D., Galley J.D., Chitchumroonchokchai C., Mace T., Suksamrarn S., Bailey M.T., Clinton S.K., Lesinski G.B. (2014). Dietary α-Mangostin, a Xanthone from Mangosteen Fruit, Exacerbates Experimental Colitis and Promotes Dysbiosis in Mice. Mol. Nutr. Food Res..

[46] Li D., Liu Q., Lu X., Li Z., Wang C., Leung C.-H., Wang Y., Peng C., Lin L. (2019). α-Mangostin Remodels Visceral Adipose Tissue Inflammation to Ameliorate Age-Related Metabolic Disorders in Mice. Aging.

[47] Mulder P., Morrison M.C., Wielinga P.Y., Van Duyvenvoorde W., Kooistra T., Kleemann R. (2016). Surgical Removal of Inflamed Epididymal White Adipose Tissue Attenuates the Development of Non-Alcoholic Steatohepatitis in Obesity. Int. J. Obes..

[48] Evans J.L., Goldfine I.D. (2013). Aging and Insulin Resistance: Just Say iNOS. Diabetes.

[49] Bumrungpert A., Kalpravidh R.W., Chitchumroonchokchai C., Chuang C.-C., West T., Kennedy A., McIntosh M. (2009). Xanthones from Mangosteen Prevent Lipopolysaccharide-Mediated Inflammation and Insulin Resistance in Primary Cultures of Human Adipocytes. J. Nutr..

[50] Bumrungpert A., Kalpravidh R.W., Chuang C.-C., Overman A., Martinez K., Kennedy A., McIntosh M. (2010). Xanthones from Mangosteen Inhibit Inflammation in Human Macrophages and in Human Adipocytes Exposed to Macrophage-Conditioned Media. J. Nutr..

[51] Kim H.M., Kim Y.M., Huh J.H., Lee E.S., Kwon M.H., Lee B.R., Ko H.-J., Chung C.H. (2017). α-Mangostin Ameliorates Hepatic Steatosis and Insulin Resistance by Inhibition C-C Chemokine Receptor 2. PLoS ONE.

[52] Rodniem S., Tiyao V., Nilbu-nga C., Poonkhum R., Pongmayteegul S. (2019). Pradidarcheep Protective Effect of Alpha-Mangostin on Thioacetamide-Induced Liver Fibrosis in Rats as Revealed by Morpho-Functional Analysis. Histol. Histopathol..

[53] Fu T., Li H., Zhao Y., Cai E., Zhu H., Li P., Liu J. (2018). Hepatoprotective Effect of α-Mangostin against Lipopolysaccharide/d-Galactosamine-Induced Acute Liver Failure in Mice. Biomed. Pharmacother..

[54] Fu T., Wang S., Liu J., Cai E., Li H., Li P., Zhao Y. (2018). Protective Effects of α-Mangostin against Acetaminophen-Induced Acute Liver Injury in Mice. Eur. J. Pharmacol..

[55] Shehata A.M., Elbadawy H.M., Ibrahim S.R.M., Mohamed G.A., Elsaed W.M., Alhaddad A.A., Ahmed N., Abo-Haded H., El-Agamy D.S. (2022). Alpha-Mangostin as a New Therapeutic Candidate for Concanavalin A-Induced Autoimmune Hepatitis: Impact on the SIRT1/Nrf2 and NF-κB Crosstalk. Plants.

[56] Nava Catorce M., Acero G., Pedraza-Chaverri J., Fragoso G., Govezensky T., Gevorkian G. (2016). Alpha-Mangostin Attenuates Brain Inflammation Induced by Peripheral Lipopolysaccharide Administration in C57BL/6J Mice. J. Neuroimmunol..

[57] McCoy J.M., Wicks J.R., Audoly L.P. (2002). The Role of Prostaglandin E2 Receptors in the Pathogenesis of Rheumatoid Arthritis. J. Clin. Investig..

[58] Liu S.-H., Chang C.-D., Chen P.-H., Su J.-R., Chen C.-C., Chaung H.-C. (2012). Docosahexaenoic Acid and Phosphatidylserine Supplementations Improve Antioxidant Activities and Cognitive Functions of the Developing Brain on Pentylenetetrazol-Induced Seizure Model. Brain Res..

[59] Lotter J., Möller M., Dean O., Berk M., Harvey B.H. (2020). Studies on Haloperidol and Adjunctive α-Mangostin or Raw *Garcinia Mangostana* Linn Pericarp on Bio-Behavioral Markers in an Immune-Inflammatory Model of Schizophrenia in Male Rats. Front. Psychiatry.

[60] Guan H., Li J., Tan X., Luo S., Liu Y., Meng Y., Wu B., Zhou Y., Yang Y., Chen H. (2020). Natural Xanthone α-Mangostin Inhibits LPS-Induced Microglial Inflammatory Responses and Memory Impairment by Blocking the TAK1/NF-κB Signaling Pathway. Mol. Nutr. Food Res..

[61] Hu Z., Wang W., Ling J., Jiang C. (2016). α-Mangostin Inhibits α-Synuclein-Induced Microglial Neuroinflammation and Neurotoxicity. Cell. Mol. Neurobiol..

[62] Yang Z., Yin Q., Olatunji O.J., Li Y., Pan S., Wang D., Zuo J. (2020). Activation of Cholinergic Anti-Inflammatory Pathway Involved in Therapeutic Actions of α-Mangostin on Lipopolysaccharide-Induced Acute Lung Injury in Rats. Int. J. Immunopathol. Pharmacol..

[63] Tao M., Jiang J., Wang L., Li Y., Mao Q., Dong J., Zuo J. (2018). *α*-Mangostin Alleviated Lipopolysaccharide Induced Acute Lung Injury in Rats by Suppressing NAMPT/NAD Controlled Inflammatory Reactions. Evid. Based Complement. Alternat. Med..

[64] Jang H.-Y., Kwon O.-K., Oh S.-R., Lee H.-K., Ahn K.-S., Chin Y.-W. (2012). Mangosteen Xanthones Mitigate Ovalbumin-Induced Airway Inflammation in a Mouse Model of Asthma. Food Chem. Toxicol..

[65] McCain J. (2013). The MAPK (ERK) Pathway. Pharm. Ther..

[66] Liu S.-H., Lee L.-T., Hu N.-Y., Huange K.-K., Shih Y.-C., Munekazu I., Li J.-M., Chou T.-Y., Wang W.-H., Chen T.-S. (2012). Effects of Alpha-Mangostin on the Expression of Anti-Inflammatory Genes in U937 Cells. Chin. Med..

[67] Shen Q., Chitchumroonchokchai C., Thomas J.L., Gushchina L.V., DiSilvestro D., Failla M.L., Ziouzenkova O. (2014). Adipocyte Reporter Assays: Application for Identification of Anti-inflammatory and Antioxidant Properties of Mangosteen Xanthones. Mol. Nutr. Food Res..

